# Circular RNA dynamics in breast-to-brain metastatic cascade

**DOI:** 10.1186/s12915-026-02666-9

**Published:** 2026-07-08

**Authors:** Adrian Szczepaniak, Jakub Karwowski, Agnieszka Bronisz, Jakub Godlewski

**Affiliations:** 1 Department of NeuroOncology, Mossakowski Medical Research Institute, Polish Academy of Sciences, Warsaw, Poland; 2 Doctoral School of Translational Medicine, Centre of Postgraduate Medical Education, Warsaw, Poland; 3 Tumor Microenvironment Laboratory, Mossakowski Medical Research Institute, Polish Academy of Sciences, Warsaw, Poland

**Keywords:** Breast cancer, Glioblastoma, Primary tumor, Metastasis, Brain metastases, Circular RNA

## Abstract

**Background:**

Breast-to-brain metastases (BTB mets) represent one of the most aggressive and clinically challenging manifestations of breast cancer. The limited efficacy of available therapies and poor patient outcomes reflect the intricate molecular and cellular processes underlying metastatic colonization of the brain. The molecular events driving this process involve extensive transcriptomic reprogramming, encompassing not only protein-coding genes but also diverse classes of non-coding RNAs. Circular RNAs (circRNAs) are a distinct class of stable, non-coding transcripts increasingly recognized as key regulators of cancer biology, yet their role in brain-tropic metastasis remains poorly defined. This study aimed to systematically profile circRNA expression across primary tumors and metastatic sites to uncover regulatory mechanisms and define site-specific circRNA expression patterns associated with BTB mets.

**Results:**

CircRNAs displayed highly site-specific expression patterns during metastatic breast cancer progression. Notably, the majority of differentially expressed circRNAs were uncoupled from changes in their linear host genes, highlighting transcriptionally-independent circularization as a regulatory mechanism. Notably, the majority of circRNAs distinguishing BTB mets from primary breast tumors and pleural effusions likewise distinguished them from primary brain tumors (glioblastoma). Analysis of the circRNA landscape revealed 42 circRNAs that are consistently deregulated and characteristic of BTB mets. Subsequent functional analysis of circRNA-microRNA interactions revealed that these circRNAs are enriched in metastatic signaling pathways and brain-related biological processes, suggesting a potential role for circRNA-mediated regulation in adaptation to the brain microenvironment. Additionally, circRNA landscapes exhibit high stability under conditions that promote either stemness or differentiation. To support data exploration, Breast circScope (circrna.netlify.app), an open-access resource that enables analysis of circRNA expression and host gene associations, along with curated visualization tools, was developed.

**Conclusions:**

Presented findings uncover a previously unrecognized dimension of post-transcriptomic regulation in metastatic breast cancer, demonstrating that circRNAs exhibit site-specific expression patterns associated with metastatic progression. These results highlight circRNAs as integral molecules in the regulatory landscape of breast cancer metastasis and provide a framework for understanding their functional contribution to brain-tropic disease.

**Supplementary Information:**

The online version contains supplementary material available at https://doi.org/10.1186/s12915-026-02666-9.

## Background

Breast-to-brain metastases (BTB mets) represent one of the most aggressive and therapeutically refractory manifestations of metastatic breast cancer. Despite improvements in systemic treatment, BTB mets remain associated with a dismal prognosis [1, 2]. While surgical resection followed by adjuvant radiotherapy extends overall survival, it rarely exceeds 2 years, varying significantly by breast cancer subtype [3–5]. Worse still, surgical resection is seldom feasible due to the presence of multiple metastatic microlesions, and radiotherapy is often ineffective, as the high doses required for tumor eradication are intolerable to the brain tissue, ultimately leaving many patients without viable treatment options.

Breast cancer epithelial cells exhibit remarkable phenotypic plasticity, allowing them to thrive and evolve under constant selective pressures–from systemic therapies to the drastically shifting microenvironmental conditions encountered along the metastatic cascade [6, 7]. As these cells disseminate from the primary tumor site, they traverse diverse biological landscapes, including transitional compartments such as malignant pleural effusion, before ultimately colonizing distant organs, including the brain [8]. Pleural effusion–the accumulation of fluid in the pleural cavity, i.e., the space between the lungs and the chest wall–often arises from tumor-induced disruption of vascular and lymphatic homeostasis [9]. A malignant pleural effusion holds a unique position within the metastatic continuum. It is neither a canonical hematogenous dissemination site nor a fully established distant metastatic niche. In metastatic breast cancer, this compartment is regarded as a temporary transit point where circulating tumor cells pause, survive, and adapt before gaining access to more protected and distal sites, such as the central nervous system [9, 10]. This intermediate microenvironment thus represents a critical phase of disease progression, capturing tumor cell populations undergoing phenotypic reprogramming and adaptation *en route* to secondary colonization of distant organs.

One of the most selective and biologically specialized sites of distant dissemination is the brain, a unique microenvironment that has been extensively characterized in the context of primary glioma biology. Within this niche, dynamic and reciprocal interactions among tumor cells, astrocytes, microglia, neurons, and the neurovascular unit critically shape malignant behavior [11, 12]. Increasing evidence indicates that similar microenvironmental networks also govern the seeding, adaptation, and progression of secondary brain metastases, including those arising from breast cancer, underscoring the central nervous system as a site imposing distinct metabolic and immunological constraints on both primary and metastatic tumors [13, 14]. Consequently, the brain represents not only a key endpoint of the metastatic cascade but also a conceptual framework for understanding how diverse tumor types adapt to neuro-specific conditions.

The reprogramming that occurs during metastatic progression is accompanied by profound molecular alterations, resulting in significant divergence between primary tumors and their corresponding metastases across genomic, epigenomic, and particularly transcriptomic levels [15, 16]. A comprehensive study by Cosgrove et al. [17] identified substantial genomic and transcriptomic heterogeneity in a large cohort of patient-matched primary breast tumors and BTB mets using RNA sequencing. The analysis revealed cancer subtype-specific gene expression networks and underscored significant transcriptomic divergence between primary and metastatic lesions [17]. Using RNA extracted from formalin-fixed, paraffin-embedded samples, the NanoString nCounter platform enabled multiplexed measurement of 252 target genes without amplification. The study identified a panel of 22 candidate genes involved in metastatic colonization but not in primary tumor development [18]. These data emphasize the existence of site-specific transcriptional programs that drive metastatic competence and microenvironmental adaptation.

Preclinical studies, including the development of ex vivo models, provide further insight into metastasis, uncovering potential molecular drivers and therapeutic targets. However, a comprehensive understanding of transcriptional changes across the multi-step metastatic process in breast cancer cells isolated from the primary tumor, pleural effusion, and BTB mets remains incomplete. This gap is particularly evident in the context of the non-coding RNA (ncRNA) transcriptome, which comprises about 98% of all transcribed RNA [19] yet remains significantly underrepresented in studies of metastatic breast cancer, including those focused on brain colonization. While most transcriptomic studies have traditionally emphasized protein-coding mRNAs, growing evidence highlights the pivotal roles of ncRNAs in gene regulation, chromatin architecture, and post-transcriptional control.

Among ncRNAs, circular RNAs (circRNAs) have recently emerged as a particularly intriguing class. Formed through a noncanonical back-splicing event, where a downstream splice donor joins an upstream splice acceptor, circRNAs create covalently closed RNA loops that are exceptionally stable and resistant to exonuclease degradation. Functionally, circRNAs act primarily through microRNA sponging, widely regarded as a key indicator of circular RNA activity, while also serving as modulators of RNA-binding proteins and, in selected contexts, as templates for cryptic peptide translation [20, 21]. Although circRNAs were first implicated in cancer biology over three decades ago [22], it was not until the advent of high-throughput RNA sequencing and specialized computational pipelines in the early 2010 s that their widespread presence and functional potential were fully appreciated [23, 24]. Since then, a growing body of evidence implicates circRNAs in fundamental steps of metastatic competence, enhancing cell migration and invasion and facilitating colonization of new microenvironments [25]. Notably, aberrant circRNA expression has also been reported in primary brain tumors, including glioblastoma, where circRNAs contribute to tumor progression and therapy resistance, further underscoring their relevance in neuro-oncology [26–28]. Moreover, some tumor and stromal cells have been reported to secrete circRNAs within exosomes, thus enabling intercellular communication that promotes the epithelial-mesenchymal transition (EMT), pre-metastatic niche formation, and organ-specific colonization [29, 30]. These considerations underscore the need for comprehensive, site-specific transcriptomic profiling of circRNAs to delineate their footprint in shaping metastatic phenotypes. Nevertheless, their specific roles in BTB mets remain elusive, representing an unexplored and promising frontier in metastasis research.

This study comprehensively profiled the transcriptomic landscape, including circRNAs, mRNAs, and long non-coding RNAs (lncRNAs), in epithelial breast cancer cells derived from three anatomically and biologically distinct sites: primary breast tumors, malignant pleural effusions, and BTB mets. As a complementary arm of the analysis, cells from the primary brain malignancy, glioblastoma, were included to provide context for a brain-specific malignant setting. Using a high-resolution microarray platform from Arraystar, biological replicates from each site were analyzed to uncover RNA expression patterns that reflect the progression of metastatic disease, with the primary aim of identifying circRNAs whose expression profiles differ significantly along the metastatic trajectory. The potential responsiveness of the circRNAome to external stimuli that induce phenotypic changes was assessed by culturing breast cancer cells under conditions designed to promote either a stem-like state or a more differentiated phenotype. To facilitate data accessibility and support collaborative research, an interactive web-based platform, *Breast circScope*, was developed to enable users to query thousands of circRNAs, mRNAs, and lncRNAs across samples.

## Results

### Comprehensive RNA profiling uncovers distinct signatures in primary and metastatic breast cancer

**Fig. 1:**
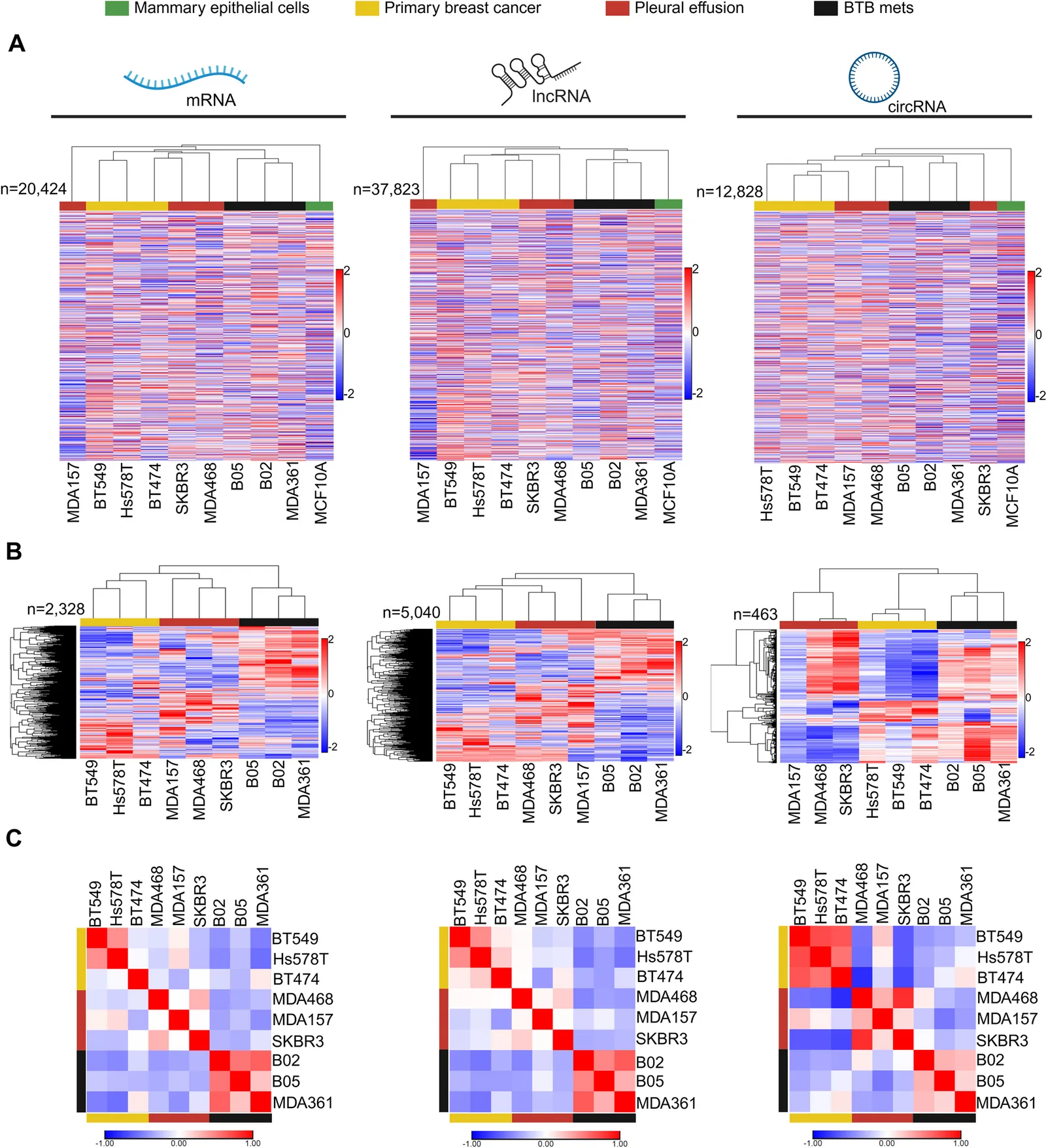
Comprehensive transcriptomic profiling of primary and metastatic breast cancer cells. Transcriptomic profiling was performed for mRNA (left), lncRNA (middle), and circRNA (right) across 10 cell lines representing distinct cellular and tumor groups: non-malignant mammary epithelial cells (*n* = 1), primary breast cancer (*n* = 3), pleural effusion (*n* = 3), and breast-to-brain metastases (BTB mets) (*n* = 3), using: **A** heatmap visualization based on all analyzed cell lines (*n* = 10); **B** test of differential expression (ANOVA, fold change > 2, *p*-value < 0.05) (*n* = 9); **C** similarity matrix (*n* = 9). The number of significantly altered transcripts is indicated in the appropriate panels

Profiling of distinct RNA subsets was performed in breast cancer cells isolated from a panoply of anatomical sites that included primary tumors from the mammary gland as well as disseminated metastatic samples from pleural effusions and the ultimate distant organ, the brain. A normal breast epithelial cell line served as a non-malignant reference (Additional file 1: Table S1). A total of 20,424 mRNAs, 37,823 lncRNAs, and 12,828 circRNAs were detected using Arraystar platforms. The log₂ ratio distributions were consistent for each RNA class across the samples upon normalization (Additional file 2: Fig. S1A). Hierarchical clustering based on this comprehensive RNA expression profiling revealed a segregation between the non-malignant mammary epithelial material and the neoplastic samples (Fig. 1A, Additional file 2: Fig. S1B). Using the ANOVA test applied to identify RNAs with differential expression across the three sites of cancer cell origin, 2,328 mRNAs, 5,040 lncRNAs, and 463 circRNAs were found to be significantly deregulated (Fig. 1B). Considering the total number of detected transcripts, the deregulation of mRNAs (11.3%) and lncRNAs (13.3%) was apparent. In contrast, circRNAs were the least-deregulated class (3.6%). Similarity matrix analysis based on circRNA expression profiles revealed a clear separation among the three cancer groups. In contrast, analysis of mRNA and lncRNA expression identified BTB mets as distinct from the remaining groups. Still, they did not distinguish between primary tumors and pleural effusion samples, indicating partially overlapping expression patterns (Fig. 1B-C). Accordingly, the expression analysis of the linear host gene for deregulated circRNAs, using hierarchical clustering and similarity matrix analyses, revealed greater divergence, resulting in less clear group separation (Additional file 2: Fig. S1C) than was observed from circRNA expression (Fig. 1B, right).

Together, these observations suggest that circRNA expression superbly captures site-specific transcriptional differences that are not readily reflected in linear transcripts. While this approach enabled the examination of circRNA expression changes along the metastatic cascade, a more granular and context-aware characterization of circRNAs profiles in BTB mets required an additional layer of biological reference. To this end, glioblastoma stem-like cells (GSCs) of transcriptomically and phenotypically diverse proneural and mesenchymal subtypes [31] were incorporated as a representative model of a primary brain tumor, along with neural progenitor cells (NPCs) as a non-malignant brain reference (Additional file 2: Fig. S1D).

### Distinct circular RNA expression defines BTB mets

**Fig. 2:**
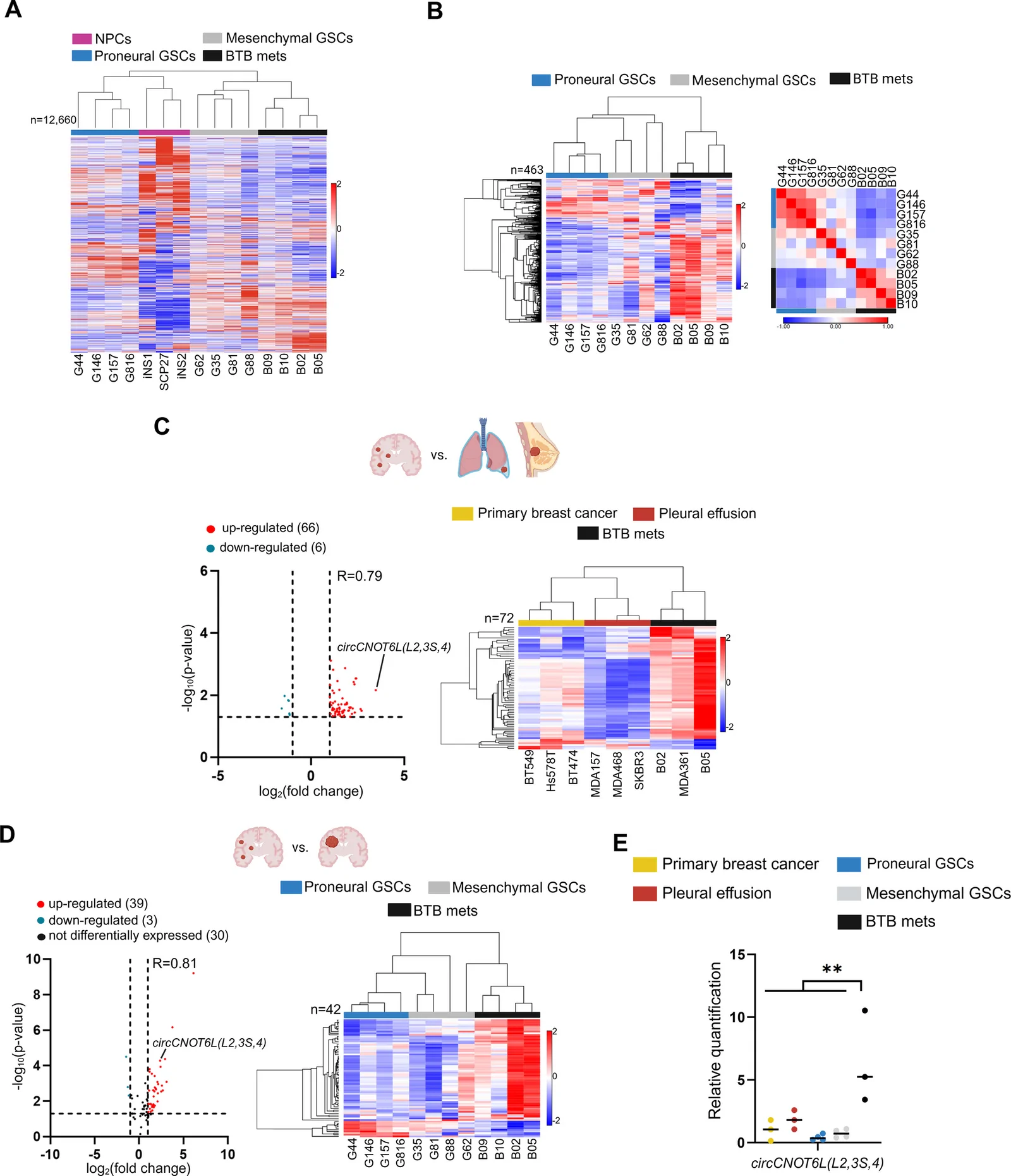
Comparative transcriptomic profiling of primary and secondary brain tumors. Transcriptomic profiling of circRNA expression was performed across 15 cell lines representing distinct cellular and tumor groups: neural progenitor cells (NPCs) (*n* = 3), glioblastoma stem-like cells (GSCs) of proneural (*n* = 4) and mesenchymal (*n* = 4) subtypes, and breast-to-brain metastases (BTB mets) (*n* = 4) using: **A** Heatmap of all detected circRNAs expression across all samples (*n* = 15); **B** Heatmap of the ANOVA-selected circular RNAs (from Fig. 1B) (left panel) and similarity matrix (right panel) (*n* = 12 cell lines); **C** Volcano plot (left panel) and heatmap (right panel) showing only differentially expressed circRNAs in BTB mets versus pleural effusions and primary breast tumors (T-test; fold change > 2, *p*-value < 0.05) (*n* = 9 cell lines); **D** Volcano plot (left panel) and heatmap (right panel) showing expression of 72 circRNAs selected in Fig. 2D between BTB mets and GSCs (T-test; fold change > 2, *p*-value < 0.05) (*n* = 12 cell lines). **E** Bar graphs showing relative qPCR expression levels of selected circular RNA in primary breast cancer (*n* = 3), pleural effusion (*n* = 3), proneural GSC (*n* = 4), mesenchymal GSCs (*n* = 4), and BTB mets (*n* = 3). The median is indicated (Mann–Whitney test, **p < 0.001. The number of significantly altered transcripts and the correlation (R) value are indicated in the appropriate panels

Profiling of circRNA expression in GSCs and NPCs was performed within a brain-centered microenvironmental model using microarray analysis. Following normalization, log₂ ratio distributions were comparable across all samples, indicating consistent circRNA detection (Additional file 2: Fig. S2A). Analysis of the complete circRNA landscape (*n* = 12,660) revealed a high degree of similarity between BTB mets and GSCs, despite their distinct tissue of origin, especially the mesenchymal subtype (Fig. 2A, Additional file 2: Fig. S2B). Notably, comparative analyses revealed upregulation of circRNAs in BTB mets relative to GSCs and NPCs (Additional file 2: Fig. S2C, D).

To relate these findings to circRNAs previously identified along the metastatic cascade, further analysis was focused on the subset of 463 circRNAs shown to be deregulated across distinct stages of breast cancer progression (Fig. 1B), and hierarchical clustering based on this circRNA set clearly separated BTB mets from both GSC subtypes (Fig. 2B). To further refine this comparison within a broader tumor context, BTB mets were contrasted with other epithelial cancer cell types resulting in the identification of 72 deregulated circRNAs in BTB mets (Fig. 2C). Notably, out of these, 42 circRNAs were also deregulated in BTB mets relative to GSCs, supporting their consistent association with the brain-tropic metastatic phenotype (Fig. 2D). Importantly, one of the most upregulated circRNAs identified in this analysis was independently validated by qPCR (Fig. 2E).

MicroRNAs were chosen as functional indices, as microRNA sponging is the most established and mechanistically validated mode of circRNA activity, allowing biological effects to be inferred indirectly from the genes they regulate. We selected 42 circRNAs that were commonly deregulated in BTB mets compared with pleural effusions and primary breast tumors, as well as between BTB mets and GSCs (Additional file 1: Table S2). Consequently, enrichment analysis of genes controlled by the prioritized microRNAs served as the foundation for assessing the functional relevance of this circRNA subset, highlighting overrepresentation of pathways associated with metastatic progression, including ErbB, Ras, MAPK, and Wnt signaling (Additional file 2: Fig. S2E). In addition, the microRNAs predicted to be sponged by the selected circRNAs were enriched in nervous system-related biological processes, including neurogenesis, nervous system development, and synaptic signaling (Additional file 2: Fig. S2F), as well as in broader remodeling of kinase-related networks (Additional file 2: Fig. S2G). Consistently, Gene Ontology Cellular Component analysis showed enrichment for brain-associated structures, including synapses, axons, and neurons (Additional file 2: Fig. S2H). Overall, this analysis highlights a distinct group of circRNAs associated with BTB mets in brain tumors. These findings support the notion that circRNA expression reflects diverse aspects of metastatic adaptation to the brain environment and provides a foundation for further mechanistic and functional investigations.

### CircRNA profile distinguished between primary and metastatic breast cancer cells

**Fig. 3:**
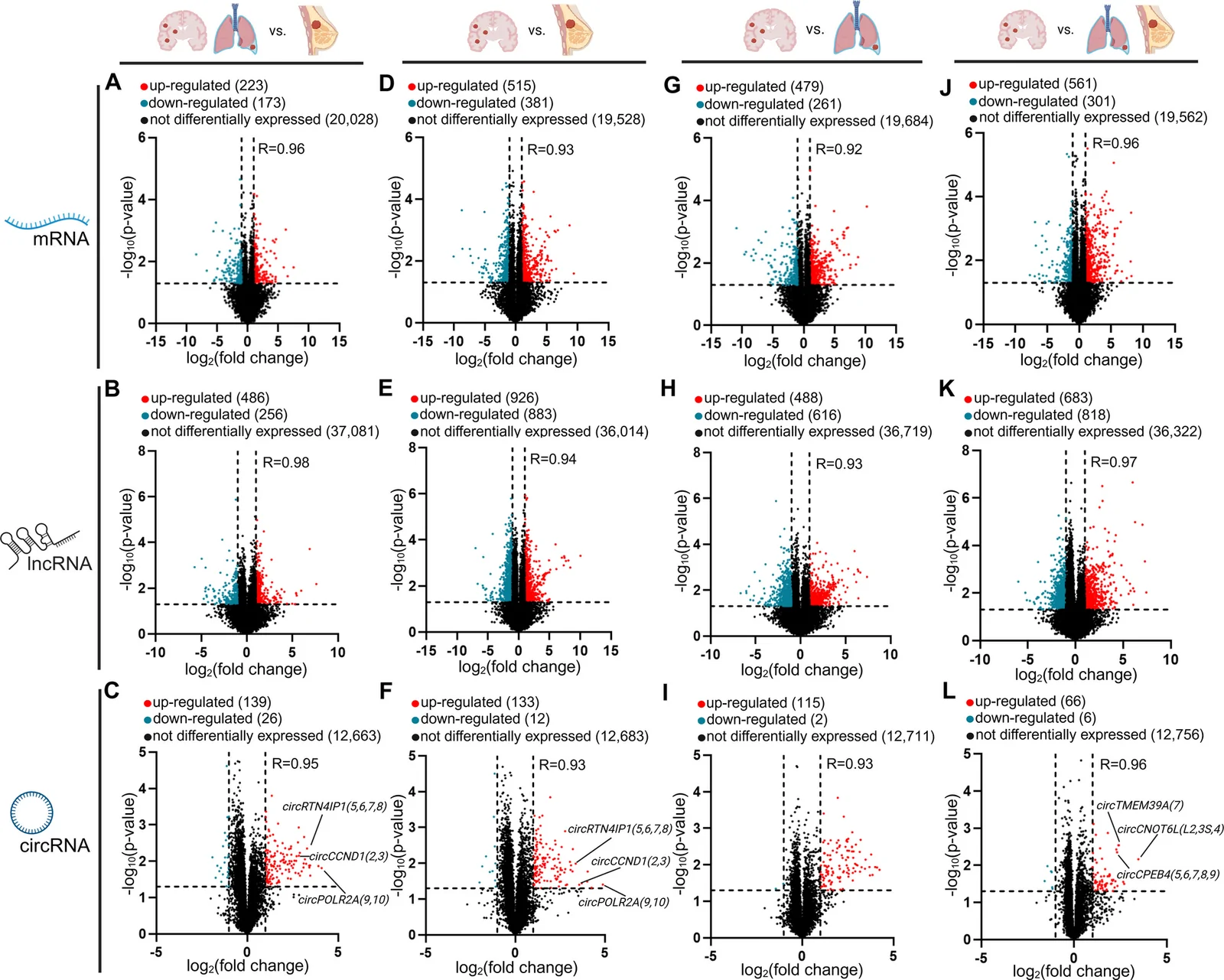
Analysis of significantly deregulated linear and circular RNAs in metastatic breast cancer. Transcriptomic comparisons were performed for mRNA (upper panel), lncRNA (middle panel), and circRNA (lower panel) expression across breast cancer cells derived from different anatomical sites, using: **A**-**C** Volcano plots comparing metastases (*n* = 6) versus primary breast cancer (*n* = 3); **D**-**F** Volcano plots comparing breast-to-brain metastases (BTB mets) (*n* = 3) versus primary breast cancer (*n* = 3); **G**-**I** Volcano plots comparing BTB mets (*n* = 3) versus pleural effusion (*n* = 3); **J**-**L** Volcano plots comparing BTB mets (*n* = 3) versus pleural effusions and primary breast cancer (*n* = 3 each). Differentially expressed transcripts were identified using a t-test (fold change > 2, *p*-value < 0.05). The number of significantly altered transcripts and the correlation (R) value are indicated in each panel

Transcriptome profiles of cancer cells stratified by their site of origin revealed molecular features associated with metastatic progression. Distinct sets of transcripts consistently distinguished cells of metastatic origin from those derived from primary breast tumors (Fig. 3A-C, D-F, Additional file 2: Fig. S3). Transcriptomic signatures also distinguished between metastatic cells originating from different anatomical sites (Fig. 3G-I), highlighting both shared and site-specific molecular adaptations associated with dissemination. In addition, BTB mets-specific analyses uncovered a distinct set of transcripts exhibiting selective deregulation in brain-tropic metastatic cells (Fig. 3J-L). These comparisons revealed a substantial number of circRNAs that were uniquely enriched in cells from a specific site of origin, suggesting a potential role for circRNAs in shaping the tissue-specific landscape of metastatic breast cancer.

Pearson correlation analysis of the entire circRNAome revealed high similarity across all groups, suggesting that only a limited fraction of circRNAs is specifically deregulated in BTB mets lesions. Interestingly, the analysis revealed that the upregulation is a significantly more prominent change of circRNAs in metastatic samples than the downregulation, contrasting with the more evenly balanced distribution of up- and downregulated transcripts observed in linear RNAs (mRNAs and lncRNAs) (Fig. 3, Additional file 2: Fig. S3).

### CircRNAs are deregulated independently of their linear host genes

**Fig. 4:**
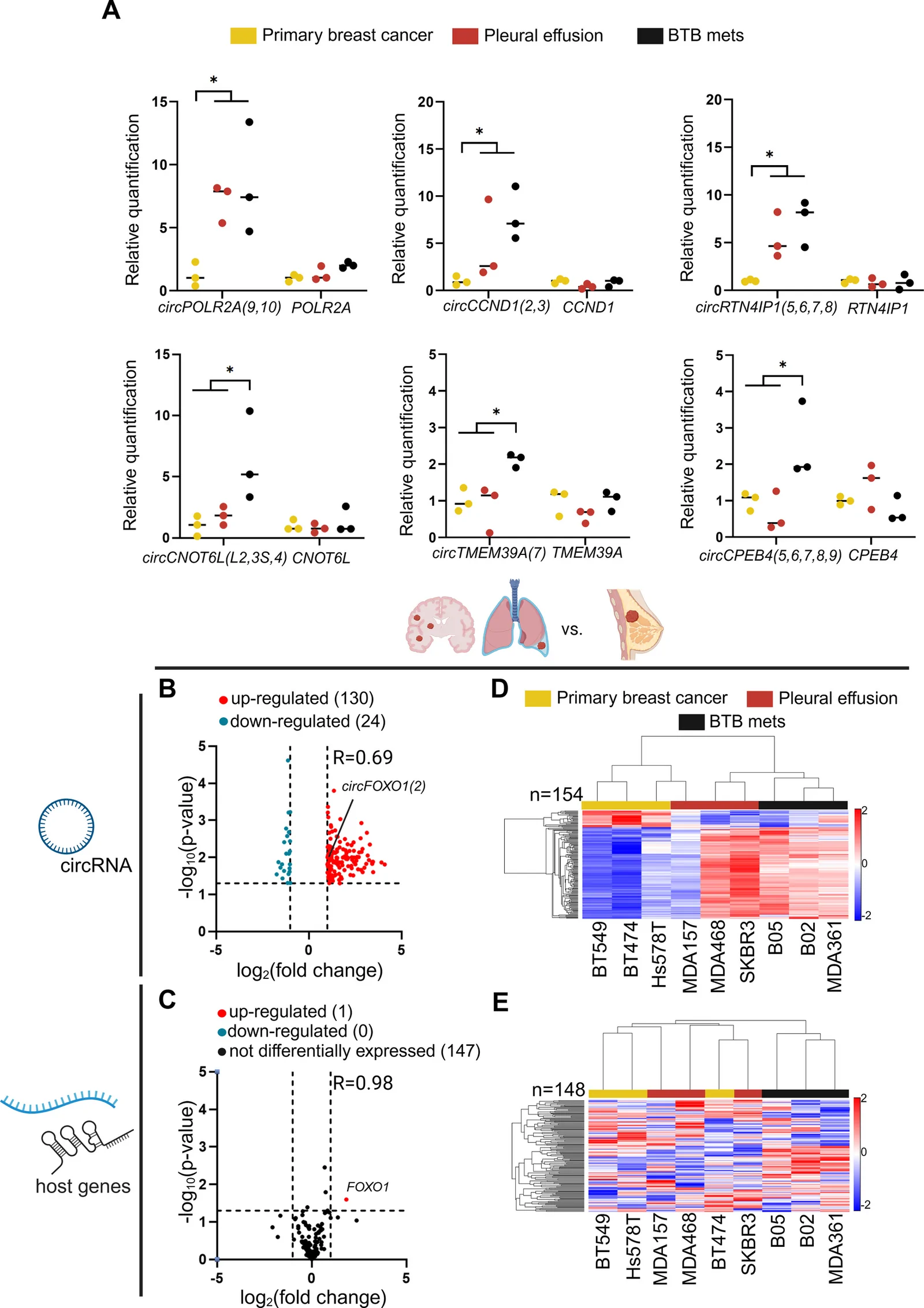
Comparative analysis of circular RNAs and their host gene expression. **A** Bar graphs showing relative qPCR expression levels of selected circular RNAs alongside their linear host gene transcripts in primary breast cancer (*n* = 3), pleural effusion (*n* = 3), and breast-to-brain metastases (BTB met) (*n* = 3). The median is indicated. (Mann-Whitney test, *p < 0.05); **B**-**C** Volcano plots displaying fold change and statistical significance of differentially expressed circRNAs and their host genes in metastases (*n* = 6) versus primary breast cancer (*n* = 3) samples (t-test; fold change > 2, *p*-value < 0.05); **D**-**E** Heatmaps illustrating the hierarchical clustering of circRNAs and their host genes across metastatic (*n* = 6) and primary breast cancer (*n* = 3) samples. Differentially expressed transcripts were identified using a t-test (fold change > 2, *p*-value < 0.05). The number of significantly altered transcripts and the correlation (R) value are indicated in the appropriate panels

To validate the microarray findings (Fig. 3C, F, I, L), the expression of the most upregulated circRNAs was assessed: *circPOLR2A(9,10)* in metastases relative to primary tumors, and *circCNOT6L(L2,3S,4)* in BTB mets relative to other epithelial cancer cells. In addition, other significantly upregulated circRNAs showing elevated expression in the presented comparisons were analyzed, including *circCCND1(2,3)*, *circRTN4IP1(5,6,7,8)*, *circTMEM39A(7)*, and *circCPEB4(5,6,7,8,9)*. Consistent with the microarray findings, the selected circRNAs were uniformly upregulated. In contrast, their host gene expression remained unchanged (Fig. 4A). These results thus suggest that specific circRNAs are associated with metastatic progression independently of their host genes.

The back-splicing process that generates circRNAs from linear transcripts remains poorly understood, making the relationship between circRNAs and their host genes particularly intriguing. Thus, the analysis of circRNAs significantly deregulated in metastatic compared to primary breast cancer, as well as between metastatic sites, along with their linear host genes, was performed, revealing that the vast majority of circRNAs were altered independently (Fig. 4B-C, Additional file 2: Fig. S4A-B, E-F). Only a few instances of coordinated deregulation of circRNAs and their linear host genes were observed, including *circFOXO1(2)* in pooled metastases versus primary breast cancer (Fig. 4B-C), as well as 7 cases in BTB mets versus primary breast cancer (Additional file 2: Fig. S4A-B) and 3 circRNAs in BTB mets versus pleural effusion (Additional file 2: Fig. S4E-F). Overall, these findings appear to be rare exceptions to the generally observed lack of correlation between circRNA and their linear host gene expression.

Hierarchical clustering based on deregulated circRNA expression distinguished cancer types by tissue of origin (Fig. 4D, Additional file 2: Fig. S4C, G). As expected, because those circRNAs’ linear host genes were not significantly deregulated, their expression demonstrated much weaker power to distinguish between the samples (Fig. 4E, Additional file 2: Fig. S4D, H). These findings point to distinct regulatory features of circRNAs that are independent of their linear host genes and thus potentially contribute to breast cancer metastasis.

### Stemness/differentiation cues shift the circRNA landscape in a cell-dependent manner

Epithelial breast cancer cells from different stages of disease, when established in culture ex vivo, displayed circRNA signatures capable of separating cancer cells from primary and metastatic sites (Figs. 1, 2, 3, and 4). To assess whether external stimuli affect such signatures, culture conditions promoting either stemness (stem) or differentiation (diff) were applied (Additional file 2: Fig. S5A). The effects of these conditions were validated by measuring the expression of cellular status markers–*E-cadherin* and *CD44*. Predictably, differentiation increased *E-cadherin* in some, but not all, cells, while *CD44* levels consistently decreased across all cell lines (Additional file 2: Fig. S5B).

**Fig. 5:**
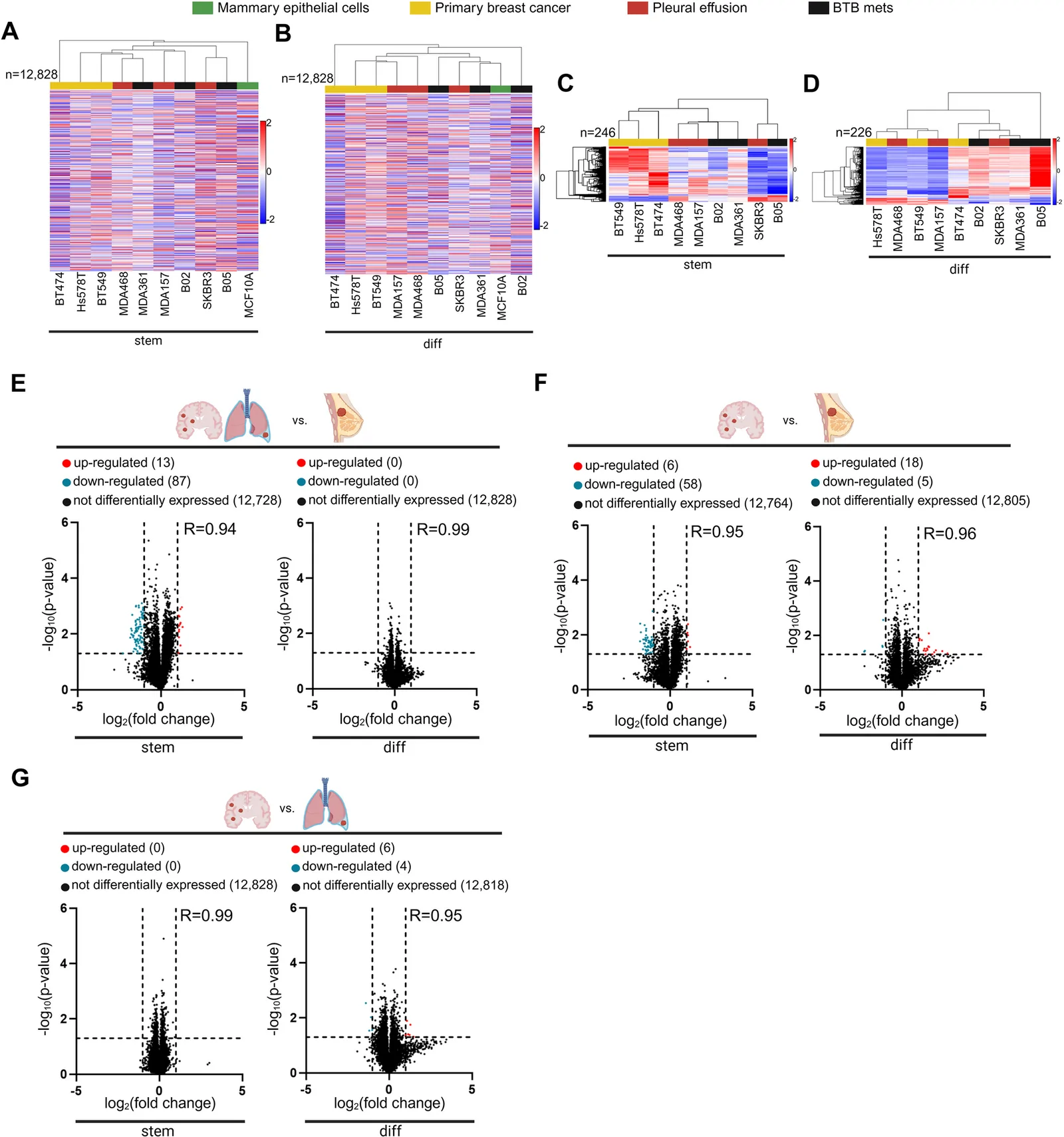
Expression patterns of circular RNAs under stemness- and differentiation-inducing culture conditions. CircRNA profiling was conducted under stemness- and differentiation-promoting conditions across three breast cancer cell groups (primary tumor, pleural effusions, and BTB metastases (BTB mets)), using: **A**-**B** Hierarchical clustering of 12,828 circRNAs detected under stemness-promoting and differentiation-promoting conditions across all samples (*n* = 10); **C**-**D** Differential expression analysis of circRNAs across the three cancer cell types under stemness and differentiation conditions (ANOVA; fold change > 2, *p*-value < 0.05); **E** Volcano plots showing circRNAs differentially expressed between metastatic samples (*n* = 6) and primary tumors (*n* = 3) under both conditions (t-test; fold change > 2, *p*-value < 0.05); **F** Volcano plots comparing circRNA expression between BTB mets (*n* = 3) and primary tumor cells (*n* = 3); **G** Volcano plots comparing circRNA expression between brain (*n* = 3) and lung pleural effusions (*n* = 3) under both conditions. The number of significantly altered transcripts and the correlation (R) value are indicated in the appropriate panels

CircRNAome analysis revealed that, under stem cell conditions, cancer cells were distinguishable from non-malignant cells (Fig. 5A), as they were in control conditions (Fig. 1A). In contrast, differentiation appeared to blur this separation (Fig. 5B), suggesting that differentiation signals override the metastasis-specific circRNA landscape.

Under control conditions, hierarchical clustering of differentially expressed circRNAs demonstrated distinct signatures for each group, separating primary breast cancer from pleural effusion and BTB mets (Fig. 1B). However, under stem conditions, the analysis revealed similarity between profiles of pleural effusion and BTB mets, distinctly separating them from primary breast cancer samples (Fig. 5C). Conversely, under diff conditions, primary breast cancer samples clustered closely with pleural effusion, both distinct from BTB mets samples (Fig. 5D).

In the global analysis, which combined all cell types and both conditions, cells responded to external stimuli individually; thus, separation based on cell type or culture condition was not apparent (Additional file 2: Fig. S5C-D). Comparison of metastases and primary breast cancer under stem conditions revealed multiple deregulated circRNAs, with a prominent pattern of downregulation (Fig. 5E), which was not as evident under control conditions (Fig. 3C). In contrast, the upregulation pattern had disappeared (Fig. 5E). Moreover, no deregulated circRNAs were detected under diff conditions. All these tendencies were broadly similar when only terminal sites (i.e., breast versus brain) of the cascade were compared (Fig. 5E-F). Interestingly, when comparing two metastases, neither responded to either external stimulus (Fig. 5G). CircRNA expression profiles were more uniform under stemness- and differentiation-promoting conditions than under control conditions, which do not promote commitment to a specific cell phenotype. In contrast, when analyzing differentially expressed circRNAs between two extreme phenotypic states within each site of origin individually, circRNA expression remained largely stable, with only a limited number of circRNAs exhibiting statistically significant changes (Additional file 2: Fig. S5E-G).

These findings suggest that the two distinct stimuli used to induce phenotypic shifts exerted only a modest influence on the circRNA landscape, highlighting that circRNAs are predominantly determined by the cell type or state rather than external conditions. Most of the observed changes were cell-type-dependent and lacked statistical robustness; however, even these subtle alterations were sufficient to influence cell clustering by site of origin. The circRNAome signature under divertible culture conditions further supports the notion that circRNA expression reflects the intrinsic cellular state linked to the cell’s origin rather than being shaped by external ex vivo stimuli. This suggests that cancer cells retain a form of tumor microenvironment "memory" that is not reprogrammed by culture conditions, underscoring the biological relevance and stability of circRNA profiles in the presented model.

### Breast circScope: an open-access platform for exploring circular and linear RNA landscapes in breast cancer

To promote broad accessibility, all data have been deposited in the GEO database and made freely available through *Breast CircScope*, an open-access web platform designed for intuitive exploration and analysis of circRNA and host gene expression data. The platform is structured into two main modules: *RNA Landscape in Breast Cancer Metastasis* and *CircRNA Expression Across Various Culture Conditions*.

Each module enables searches by gene name, circRNA ID, or genomic position, providing comprehensive outputs that include general annotations, normalized expression values, predicted microRNA-binding sites, and the probe sequences used in the microarray. An integrated Advanced Search feature facilitates rapid interrogation of pre-analyzed datasets. The *RNA Landscape in Breast Cancer Metastasis* module allows investigation of differential expression patterns of mRNAs, lncRNAs, and circRNAs across breast cancer samples derived from distinct anatomical sites. In parallel, the *CircRNA Expression Across Various Culture Conditions* module enables the assessment of circRNA expression dynamics in response to stemness- and differentiation-promoting conditions across multiple cancer cell types.

Both modules offer advanced tools, including ranked lists of the top 10 upregulated and downregulated transcripts, full transcriptome downloads, and visualizations such as heatmaps, similarity matrices, and volcano plots. A dedicated Comparison section, unique to the *Culture Conditions* module, supports identification of circRNAs exhibiting condition-specific expression changes within the same cell line. By integrating open-access datasets with a user-friendly interface, Breast CircScope serves as a comprehensive resource for exploring, comparing, and interpreting RNA and circRNA expression patterns in breast cancer, with a focus on metastatic progression and cellular responses to environmental stimuli.

## Discussion

Breast cancer metastasis remains one of the most intractable challenges in modern medicine and biomedical research. Consequently, there is growing interest in uncovering the molecular mechanisms that underlie metastatic spread and identifying the key drivers of this process. This study comprehensively profiled circRNAs and linear transcripts, including mRNAs and lncRNAs, using an ex vivo model of breast cancer metastasis encompassing primary tumors, pleural effusions, and brain-tropic metastases. In addition, circRNA expression was analyzed in a model of a primary brain tumor, glioblastoma, providing a brain-centered reference for comparison. Particular emphasis was placed on circRNAs, a recently characterized class of non-coding RNAs increasingly implicated in cancer progression and metastasis.

Transcriptomic profiling of breast cancer cells from primary tumors, malignant pleural effusions, and BTB mets revealed that circRNAs display distinct expression signatures throughout the metastatic cascade. To date, high-throughput screens have identified hundreds of circRNAs whose expression shifts in brain-tropic breast cancer models [32]. For example, *circBCBM1(3,4,5,6,7,8,9,10,11)* was significantly upregulated in BTB mets compared with parental cell lines [33] Other studies have documented that circRNAs function as either oncogenes (e.g., *circEZH2(2,3, L4)* [34]) or tumor suppressors (e.g., *circCCDC85A(2)* [35]) in breast cancer, regulating proliferation, invasion, and metastatic potential. The data reaffirm these findings and extend them by identifying additional brain-enriched circRNAs with similar regulatory potential, indicating that the metastatic microenvironment and selective pressures help shape a unique circRNA landscape. Notably, some circRNAs were consistently upregulated in metastases relative to the primary tumor, suggesting potential pro-metastatic circRNA candidates. The covalently closed-loop structure of circRNAs renders them resistant to exonuclease-mediated degradation, thereby increasing their stability compared to linear RNAs. These findings collectively indicate that circRNAs are dynamically regulated during metastatic progression and exhibit site-specific expression patterns, with notable molecular stability, suggesting their potential involvement in the biology of breast cancer metastasis.

Extending circRNA profiling to a brain-centered framework provided insight into circRNA regulation in BTB mets within the broader landscape of brain tumors and neural cell types. By incorporating GSCs and NPCs, metastatic circRNA expression was juxtaposed with both malignant and non-malignant brain-resident states. Overall, BTB mets exhibited substantial similarity to GSCs in global circRNA expression, despite their different tissue of origin. Notably, this similarity was most pronounced when compared with the mesenchymal GSC subtype, likely reflecting that breast cancer cells colonizing the brain have typically undergone EMT and adopted a more invasive, mesenchymal-like state. EMT has been extensively implicated in metastatic dissemination and organ colonization, including brain metastasis, and is associated with transcriptional plasticity and increased adaptability to foreign microenvironments [36]. Previous studies also have shown that BTB mets often display mesenchymal features and engage stemness-associated and glial-related programs that overlap with those observed in mesenchymal glioblastoma cells [37–39].

Although BTB mets showed an overall circRNA profile similar to that of brain tumor cells, this resemblance did not hold for all circRNAs. When the analysis focused on a defined set of circRNAs previously linked to breast cancer progression, BTB mets separated clearly from primary brain tumors. This suggests that, alongside brain-adaptive changes, metastatic cells retain a distinct circRNA signature associated with their breast/epithelial origin and metastatic evolutionary history. Taken together, the data support a “hybrid” regulatory state: BTB mets adopt selected brain-associated features while maintaining some lineage-specific traits, consistent with prior transcriptomic and chromatin-based studies showing that brain metastases combine organ-adaptation programs with features inherited from the primary tumor [40, 41].

Among the most critical features of metastasis are enhanced invasion, motility, and migration-processes tightly regulated by a variety of signaling networks, many of which are susceptible to circRNAs, whose roles in promoting metastatic traits are increasingly apparent. CircRNAs preferentially deregulated in BTB mets relative to GSCs and other epithelial cancer types define a subset associated with brain-tropic metastatic behavior. Functional analysis in this study was framed within the circRNA-microRNA-mRNA regulatory axis, in which deregulated circRNAs are predicted to bind specific microRNAs, thereby reducing their availability. Because microRNAs normally repress the expression of their target mRNAs, circRNA-mediated sequestration may increase the expression of downstream mRNA targets. These mRNAs encode components of key signaling pathways that regulate cell migration, invasion, and survival, thereby providing a mechanistic link between circRNA deregulation and pro-metastatic cellular behavior. Consistent with this framework, functional inference based on predicted microRNA interactions linked these circRNAs to key oncogenic pathways, including ErbB, Ras, MAPK, and Wnt signaling, which are known to support metastatic colonization of the brain[42–47]. In parallel, the associated microRNA networks were enriched for biological processes related to nervous system development and function, in line with accumulating evidence that BTB mets actively engage neural signaling programs and establish functional interactions with resident brain cells to support survival and outgrowth [14, 37, 48]. Taken together, these findings suggest that circRNAs actively participate in regulatory circuits that couple oncogenic signaling with brain-specific cues, thereby contributing to metastatic adaptation within the brain microenvironment.

One of the central findings is that differentially expressed circRNAs in metastatic breast cancer are largely uncoupled from the expression of their linear host genes. This observation echoes prior findings. For instance, *circSMARCA5(15,16)* levels were reduced in breast cancer despite *SMARCA5* mRNA remaining unchanged, suggesting a transcription-independent regulatory mechanism, perhaps via altered splicing efficiency or circularization dynamics [49]. These mechanisms likely operate widely across metastatic niches, as*,* for instance, in glioblastoma, 41.84% of differentially expressed circRNAs were associated with changes in host gene expression [50], indicating that the majority were regulated independently. This highlights variability in circRNA-host gene coupling across malignancies and underscores the prevalence of host gene-independent circRNA deregulation. The independence of circRNA expression from linear host genes is further supported by evidence that alterations in the splicing machinery, such as dysregulation of spliceosomal components or RNA-binding proteins like QKI or ADAR1, contribute to this regulatory divergence [51, 52]. Together, these findings reinforce the concept that circRNAs constitute a distinct regulatory layer within the metastatic transcriptome. Crucially, such independence from host gene expression highlights a limitation of mRNA- or lncRNA-focused profiling approaches, which overlook circRNA-specific changes. From a translational perspective, the distinct expression patterns of circRNAs provide informative molecular features relevant to metastasis and its regulation.

It is well established that linear transcripts, particularly mRNAs and lncRNAs, play an active role in regulating the dynamic balance between stemness and differentiation [53, 54]. This raises the question of whether circRNAs are similarly responsive to external cues that influence cell state. However, comparative analysis of circRNA expression under stemness-promoting and differentiation-inducing conditions revealed only modest changes, with relatively few circRNAs exhibiting statistically significant differential expression. Notably, circRNA profiles appeared highly tissue-specific and largely unaffected by transient shifts in cell state, suggesting that their expression is more shaped by the metastatic site or cellular origin than by dynamic transcriptional programs. This stability is consistent with previous findings demonstrating the remarkable resistance of circRNAs to exonuclease-mediated degradation in cells and extracellular fluids [55, 56]. This inherent stability distinguishes circRNAs from linear transcripts and highlights their suitability as stable molecular features, particularly in liquid biopsy-based approaches for cancer detection and longitudinal disease assessment.

One of the key outcomes of this study is the development of Breast circScope (circrna.netlify.app), an open-access platform compiling circRNA, mRNA, and lncRNA expression data from primary and metastatic breast cancer samples. Unlike general databases such as CircBase (circbase.org) [57], CIRCpedia v2 (picb.ac.cn/rnomics/circpedia) [58], and CircAtlas (circatlas.biols.ac.cn) [59], which aggregate circRNA data across various species and tissues, Breast circScope is tailored for metastasis-focused research. This resource aligns with broader efforts to promote transparency and accessibility in cancer genomics, thus supporting more efficient data repurposing. Updates to the circScope platform would incorporate accumulating datasets–such as circRNA profiles associated with various therapeutic regimens or drug-resistant tumors–thereby establishing it as a continuously evolving repository. To the best of the authors’ knowledge, Breast circScope is the first publicly available database dedicated to circRNAs in metastatic breast cancer, offering insights into their spatial expression and potential roles in disease progression. Looking ahead, expanding *Breast circScope* to include single-cell circRNA profiles and relevant clinical metadata would significantly enhance the database’s utility for functional studies and translational research.

## Conclusions

In conclusion, this study extends breast cancer transcriptomics by defining the circRNA landscape along the metastatic progression cascade and in a brain-centered tumor context. It demonstrates that circRNAs form stable and stage-specific expression programs in primary tumors, peripheral metastases, and brain lesions, reflecting key aspects of metastatic evolution and organ-specific adaptation. Notably, most dysregulated circRNAs exhibit expression patterns decoupled from their linear host genes, underscoring a distinct regulatory layer with potential functional relevance in metastasis. The pronounced stability of circRNAs under external in vitro perturbations further highlights their robustness and suitability as reliable molecular readouts. Beyond these biological insights, the establishment of Breast circScope provides a valuable, open-access resource that integrates circRNA, mRNA, and lncRNA expression across metastatic contexts, enhancing data accessibility, reproducibility, and discovery. Collectively, these findings position circRNAs as a critical yet underexplored molecules of the metastatic transcriptome and lay a strong foundation for future mechanistic and functional investigations into organ-specific metastatic progression.

## Methods

### Cell culture

Non-commercially available human biological samples: patient-derived glioblastoma cell lines (GSCs) and breast-to-brain metastatic (BTB mets) cancer cell lines are the property of the Mossakowski Medical Research Center, Polish Academy of Sciences, based on Material Transfer Agreement (MTA) between MMRI PAS and Brigham and Women’s Hospital (BWH) as documented by Massachusetts General Hospital Uniform Biological MTA Implementing Letter (date of certification 07/09/2019). Primary cells were obtained by dissociation of brain tumor specimens of patients with glioblastoma and BTB mets diagnosis after surgical resection. De-identified samples were obtained with patient consent for harvesting, research procedures, and storage, and were approved by the Institutional Review Board (or Ethics Committee) of BWH (protocol code 2013P001236, date of approval 24.06.2013). Neural progenitor cells were obtained as previously described [60]. All other cell lines were purchased from ATCC.

BT549, Hs578T, BT474, MDA468, MDA157, SKBR3, B02, B05, B09, B10, and MDA361 were grown in Dulbecco's modified Eagle's medium (DMEM) (Gibco) supplemented with 10% (vol/vol) fetal bovine serum (FBS) (Gibco) and 1% (vol/vol) antibiotic/antymycotic solution (Capricorn). The MCF10A cell line was grown in HuMEC Ready Medium (Thermo Fisher Scientific). Mycoplasma testing was performed routinely using the MycoBlue Mycoplasma Detector (Vazyme). GSCs and NPC were cultured as neurospheres using Neurobasal medium (Gibco) supplemented with 1% (v/v) glutamine (Gibco), 2% (v/v) B27 Supplement minus vitamin A (Gibco), 20 ng/mL EGF (Gibco), 20 ng/mL bFGF (Gibco), and 1% (v/v) antibiotic/antymycotic solution using Nunclon™ Sphera™ ultralow attachment plates/flasks (Thermo Fisher Scientific).

For stemness‐promoting conditions, epithelial cells were cultured on Nunclon™ Sphera™ plates in DMEM supplemented with 30% (v/v) F-12 Supplement (Sartorius), 2% (v/v) B-27 Supplement minus vitamin A, 20 ng/mL EGF, 20 ng/mL bFGF, and 1% (v/v) antibiotic/antymycotic solution. For differentiation-promoting conditions, cells were plated on Tissue Culture (TC)-treated surface flasks (TPP) in DMEM supplemented with 30% (v/v) F12 Supplement, 2% (v/v) fetal bovine serum (FBS), 1 µM all-trans-retinoic acid (Sigma-Aldrich), and 1% (v/v) antibiotic/antymycotic solution. Before treatment, all cells were synchronized by incubation in plain DMEM for 16 h.

### RNA isolation

Total RNA was extracted using a standard TRIzol protocol (Invitrogen). To eliminate DNA, 10 μg of total RNA was incubated for 10 min at 37 °C with DNase I (New England Biolabs), followed by column purification using the Monarch Spin RNA Cleanup Kit (New England Biolabs). The RNA quantity and quality were measured using a NanoDrop One (Thermo Fisher Scientific).

### Gene microarray

Arraystar Human LncRNA Arrays V5 were designed to profile lncRNAs and the entire set of protein-coding mRNAs systematically. Sample labeling and array hybridization were performed according to the Agilent One-Color Microarray-Based Gene Expression Analysis protocol (Agilent Technology) with minor modifications. Each RNA sample was amplified and transcribed into fluorescent cRNA spanning the full length of the transcripts without 3'-bias, using a random-priming method (Arraystar Flash RNA Labeling Kit, Arraystar). The labeled cRNAs were purified by the RNeasy Mini Kit (Qiagen). The concentration and specific activity of the labeled cRNAs (pmol Cy3/μgcRNA) were measured by NanoDrop ND-1000. 1 μg of each labeled cRNA was fragmented by adding 5 μl 10 × Blocking Agent and 1 μl of 25 × Fragmentation Buffer, then heated the mixture at 60 °C for 30 min, finally 25 μl 2 × GE Hybridization buffer was added to dilute the labeled cRNA. 50 μl of hybridization solution was dispensed into the gasket slide, which was then assembled onto the LncRNA expression microarray slide. The slides were incubated for 17 h at 65 °C in an Agilent Hybridization Oven. The hybridized arrays were washed, fixed, and scanned using the Agilent DNA Microarray Scanner (part number G2505C). Agilent Feature Extraction software (version 11.0.1.1) was used to analyze acquired array images. Quantile normalization and subsequent data processing were performed using GeneSpring GX v12.1 (Agilent Technologies). The accession number for the array data is GEO: GSE302028.

### CircRNA microarray

ArrayStar Inc. performed the Human circRNA Array V2. Sample labeling and array hybridization were performed per the manufacturer's protocol (Arraystar Inc.). Total RNAs were digested with RNase R (Epicentre, Inc.) to remove linear RNAs and enrich circular RNAs. Then, the enriched circular RNAs were amplified and transcribed into fluorescent cRNA using a random-priming method (Arraystar Super RNA Labeling Kit; Arraystar). The labeled cRNAs were purified by the RNeasy Mini Kit (Qiagen). The concentration and specific activity of the labeled cRNAs (pmol Cy3/μg cRNA) were measured by NanoDrop ND-1000. 1 μg of each labeled cRNA was fragmented by adding five μl of 10 × Blocking Agent and one μl of 25 × Fragmentation Buffer. The mixture was then heated at 60 °C for 30 min. Finally, 25 μl of 2 × Hybridization buffer was added to dilute the labeled cRNA. 50 μl of hybridization solution was dispensed into the gasket slide, which was then assembled with the circRNA expression microarray slide. The slides were incubated for 17 h at 65 °C in an Agilent Hybridization Oven. The hybridized arrays were washed, fixed, and scanned using the Agilent Scanner G2505C. Scanned images were imported into Agilent Feature Extraction software to extract raw data. Statistical significance was assessed using an unpaired t-test. CircRNAs with fold changes of > 2 and *p*-values of < 0.05 were selected as significantly differentially expressed. The array data have been deposited in GEO under accession numbers GSE302027 and GSE314175.

### Quantitative PCR

Reverse transcription was performed using High-Capacity RNA-to-cDNA™ Kit (Thermo Fisher Scientific), and qPCR was performed using SYBR Green master mix (Thermo Fisher Scientific). Amplification was performed using the QuantStudio 5 Real-Time PCR System (Applied Biosystems), and the software determined *C*t thresholds. Expression was quantified using the ΔΔ*C*t method, with 18S ribosomal RNA as the reference gene. Used primers are listed below:

- *CCND1* for: TCTACACCGACAACTCCATCCG
- *CCND1* rev: TCTGGCATTTTGGAGAGGAAGTG
- *circCCND1(2,3)* for: GAGGCGGAGGAGAACAAACA
- *circCCND1(2,3)* rev: CGGTCCAGGTAGTTCATGGC
- *RTN4IP1* for: CGTTCGATTCTCTGGCGTTG
- *RTN4IP1* rev: CTCACTTTCACCCCCTCCAC
- *circRTN4IP1(5,6,7,8)* for GAGTCCATTATCGCTGGGCA
- *circRTN4IP1(5,6,7,8)* rev: GCAAAAGTACCAACTCCGCC
- *POLR2A* for: GAGCGGGGTGAAGTGATGAA
- *POLR2A* rev: GCTTGTAAGGGCCACTGTCT
- *circPOLR2A(9,10)* for: CGGCCTGTCATGGGTATTGT
- *circPOLR2A(9,10)* rev: TTTTGTGCAGAGTTGGCTGC
- *TMEM39A* for: TCTGTAAAGTTGTCGCCGCT
- *TMEM39A* rev: CCACCTGTAGTTGCAACCCA
- *circTMEM39A(7)* for: AAATCAGCTGCTCACCTGGG
- *circTMEM39A(7)* rev: AGCGCATGTCATAGTACTGGG
- *CNOT6L* for: AGAAATCTCGGGTAGAGTGCG
- *CNOT6L* rev: AGCTTGGCAATATCAGGTGGA
- *circCNOT6L(L2,3S,4)* for: ACCAGCAGAACTAGGAAACA
- *circCNOT6L(L2,3S,4)* rev: TGGCATCCCTATTAGTCTTTC
- *CPEB4* for: AGCTCTGCCTTTGCACCTAA
- *CPEB4* rev: AGAGAGCTATCGGATCCTGG
- *circCPEB4(5,6,7,8,9)* for: ATCAGTGCCCGCTTTGTTCA
- *circCPEB4(5,6,7,8,9)* rev: CCACGGCCATCATCCAAGAA
- *E-cadherin* for: CACCACGTACAAGGGTCAGG
- *E-cadherin* rev: ATTGGGGGCATCAGCATCAG
- *CD44* for: AAGGTGGAGCAAACACAACC
- *CD44* rev: CTTCTGCCCACACCTTCTTC
- *18S* for: AACTTTCGATGGTAGTCGCCG
- *18S* rev: CCTTGGATGTGGTAGCCGTTT

### CircRNAs nomenclature

CircRNAs described in this study were named in accordance with the recently established circRNA nomenclature guidelines to ensure clarity, consistency, and compatibility with community standards [61]. Briefly, circRNAs were annotated based on their genomic coordinates, host gene, and back-splice junction, following the recommended formatting style.

### Software and visualization

Statistical analyses and graph generation were performed using GraphPad Prism.

Heatmaps and hierarchical clustering were generated using Morpheus (https://software.broadinstitute.org/morpheus), with Pearson distance as the metric and complete linkage clustering based on z-score normalization. BioRender was used for schematic and illustrative visualizations.

### Comparative analysis of circular RNAs and their linear host genes

To evaluate whether differential expression was shared between circRNAs and their corresponding linear host genes, datasets GSE302027 and GSE302028 were analyzed. A total of 463 circRNAs (Fig. 1B, left panel) and 367 linear host genes were included. The difference in counts is attributable to the generation of multiple circRNAs from individual host genes, with 17 circRNAs lacking host-gene probes. Likewise, the differences observed in the corresponding analyses presented in Figs. 4 and S4 reflect either multiple circRNAs derived from a single host gene or the absence of expression data for the linear host genes.

### Functional analysis

Functional analysis of selected circRNAs was performed using their top five predicted microRNA binding partners. microRNAs with predicted binding sites on at least two circRNAs were selected, and their target genes were identified using miRWalk. Functional enrichment analyses (KEGG and GO Biological Process, Molecular Function, and Cellular Component) were conducted using ShinyGO.

### Breast circScope

The web application analyzed in this study is publicly available at circrna.netlify.app and is deployed on the Netlify platform, which provides cloud-based continuous deployment, automated build pipelines, and globally distributed content delivery, ensuring reliable access, scalability, and reproducibility without requiring local installation.

## Supplementary Information

Additional file 1: Tables S1-2. Table S1- [Classification of cell lines utilized in the experiments]. Table S2 - [Circular RNAs commonly deregulated in BTB mets compared with all other cancer cell lines, together with the top five predicted miRNA interactors for each circRNA].

Additional file 2: Figures S1-5. Fig. S1 - [Comparative Transcriptomic Profiling of Primary and Metastatic Breast Cancer Across RNA Classes]. Fig. S2 - [Comparative Transcriptomic Profiling of Primary and Secondary Brain Tumors]. Fig. S3 - [Venn diagrams illustrating the number of differentially expressed mRNAs, lncRNAs, and circRNAs across pairwise comparisons]. Fig. S4 - [Analysis of Expression Correlation Between Circular RNAs and Their Linear Host Genes]. Fig. S5 - [Expression of Circular RNAs in Breast Cancer Cells from Different Anatomical Sites Cultured Under Stemness- and Differentiation-Promoting Conditions].

Additional file 3: The quantitative data underlying the figures.

## Data Availability

Microarray-derived data are publicly available in the GEO repository with accessions: GSE302028 [62], GSE302027 [63], and GSE314175 [64] and through the Breast circScope platform (https://circrna.netlify.app/). The quantitative data underlying the figures are provided in the Additional file 3 in the supplementary materials.

## Abbreviations

BTB mets: Breast-to-brain metastases
circRNAs: Circular RNAs
ncRNA: Non-coding RNA
EMT: Epithelial-mesenchymal transition
GSCs: Glioblastoma stem-like cells
NPCs: Neural progenitor cells
lncRNAs: Long non-coding RNAs
stem: Stemness
diff: Differentiation
