# Supplementary material for: Circular RNA dynamics in breast-to-brain metastatic cascade
Source: BMC Biol. 2026 Jul 8;24:207. doi: 10.1186/s12915-026-02666-9 (PMC13632408; doi:10.1186/s12915-026-02666-9)
Supplement: Supplementary file 1 [file 12915_2026_2666_MOESM1_ESM.docx]

**Table S1. Classification of cell lines utilized in the experiments**

| **Cell line** | **Site of origin** | **Subtype** | **Source** |
| --- | --- | --- | --- |
| BT549 | Primary - Breast tissue | TNBC (Basal-like) | ATCC |
| Hs578T | Primary - Breast tissue | TNBC (Basal-like) | ATCC |
| BT474 | Primary - Breast tissue | Luminal B (ER+,PR+,HER2+) | ATCC |
| MDA468 | Metastatic - Pleural effusion | TNBC (Basal-like) | ATCC |
| MDA157 | Metastatic - Pleural effusion | TNBC (Basal-like) | ATCC |
| SKBR3 | Metastatic - Pleural effusion | HER2+ | ATCC |
| B02 | Metastatic - Brain | TNBC (Basal-like) | PMID: 33658202 |
| B05 | Metastatic - Brain | TNBC (Basal-like) | PMID: 33658202 |
| B09 | Metastatic - Brain | TNBC (Basal-like) | PMID: 33658202 |
| B10 | Metastatic - Brain | TNBC (Basal-like) | PMID: 33658202 |
| MDA361 | Metastatic - Brain | Luminal B (ER+,PR-,HER2+) | ATCC |
| G44 | Primary – Brain (Glioblastoma) | Proneural | PMID: 33328224 |
| G146 | Primary – Brain (Glioblastoma) | Proneural | PMID: 33328224 |
| G157 | Primary – Brain (Glioblastoma) | Proneural | PMID: 33328224 |
| G816 | Primary – Brain (Glioblastoma) | Proneural | PMID: 33328224 |
| G81 | Primary – Brain (Glioblastoma) | Mesenchymal | PMID: 33328224 |
| G35 | Primary – Brain (Glioblastoma) | Mesenchymal | PMID: 33328224 |
| G62 | Primary – Brain (Glioblastoma) | Mesenchymal | PMID: 33328224 |
| G88 | Primary – Brain (Glioblastoma) | Mesenchymal | PMID: 33328224 |
| MCF10A | Normal Breast | Epithelial | ATCC |
| iNS1 | Normal Brain | Neural progenitor | PMID: 30683859, 33328224 |
| iNS2 | Normal Brain | Neural progenitor | PMID: 30683859, 33328224 |
| SCP27 | Normal Brain | Neural progenitor | PMID: 30683859, 33328224 |

**Table S2. Circular RNAs commonly deregulated in BTB mets compared with all other cancer cell lines, together with the top five predicted miRNA interactors for each circRNA (MRE1-5).** Underlined miRNAs are shared by at least two circRNAs and were used for functional analysis.

| CircRNA | Regulation | MRE1 | | MRE2 | MRE3 | MRE4 | MRE5 |
| --- | --- | --- | --- | --- | --- | --- | --- |
| *circZNF516(5,6)* | up | hsa-miR-762 | hsa-miR-6812-5p | | hsa-miR-6885-5p | hsa-miR-5008-3p | hsa-miR-5001-5p |
| *circACVR2A(3,4,5,6)* | up | hsa-miR-511-5p | hsa-miR-608 | | hsa-miR-6783-3p | hsa-miR-4675 | hsa-miR-4651 |
| *circNIPSNAP3B(2,3,4,5)* | up | hsa-miR-548p | hsa-miR-570-3p | | hsa-miR-548f-5p | hsa-miR-7703 | hsa-miR-5700 |
| *circLRP6(2)* | up | hsa-miR-128-2-5p | hsa-miR-640 | | hsa-miR-128-1-5p | hsa-miR-3065-5p | hsa-miR-5695 |
| *circGLIS2(2,3)* | up | hsa-miR-6889-5p | hsa-miR-4538 | | hsa-miR-185-3p | hsa-miR-6803-5p | hsa-miR-6752-5p |
| *circALDH1L1(10,11,12,13,14,15,16)* | up | hsa-miR-6792-3p | hsa-miR-7162-5p | | hsa-miR-1226-3p | hsa-miR-4640-5p | hsa-miR-3918 |
| *circCNOT6L(L2,3S,4)* | up | hsa-miR-3692-5p | hsa-miR-3529-5p | | hsa-miR-4642 | hsa-miR-324-5p | hsa-miR-6883-3p |
| *circFAM126A(3,4,5,6,7)* | up | hsa-miR-6509-3p | hsa-miR-3192-5p | | hsa-miR-4490 | hsa-miR-1261 | hsa-miR-324-3p |
| *circFAM126A(2,3,4,5,6,7)* | up | hsa-miR-6509-3p | hsa-miR-3192-5p | | hsa-miR-548ax | hsa-miR-4680-5p | hsa-miR-548ao-5p |
| *circAS3MT(8,9)* | up | hsa-miR-593-5p | hsa-miR-29a-3p | | hsa-miR-29b-3p | hsa-miR-500b-3p | hsa-miR-7843-5p |
| *ciBLNK(3)* | up | hsa-miR-3160-5p | hsa-miR-3685 | | hsa-miR-141-5p | hsa-miR-6885-3p | hsa-miR-578 |
| *circATP5G1(4,5)* | up | hsa-miR-6876-5p | hsa-miR-4476 | | hsa-miR-3065-3p | hsa-miR-338-3p | hsa-miR-647 |
| *circPANX2(3)* | up | hsa-miR-362-3p | hsa-miR-3177-5p | | hsa-miR-574-3p | hsa-miR-2276-5p | hsa-miR-9851-3p |
| *circEPHA3(3)* | up | hsa-miR-4700-3p | hsa-miR-96-5p | | hsa-miR-1236-3p | hsa-miR-4731-3p | hsa-miR-1537-5p |
| *ciENSG00000263620(4)* | up | hsa-miR-3145-5p | hsa-miR-519d-5p | | hsa-miR-8064 | hsa-miR-1469 | hsa-miR-6875-3p |
| *circIP6K2(4,5)* | up | hsa-miR-4778-3p | hsa-miR-4659b-3p | | hsa-miR-4659a-3p | hsa-miR-1248 | hsa-miR-647 |
| *ciSTAM(13)* | up | hsa-miR-4793-3p | hsa-miR-4325 | | hsa-miR-20a-3p | hsa-miR-6500-3p | hsa-miR-770-5p |
| *circVAT1L(2,3)* | up | hsa-miR-6866-3p | hsa-miR-8073 | | hsa-miR-494-5p | hsa-miR-3689b-3p | hsa-miR-3689c |
| *ciCNPY3(3)* | up | hsa-miR-6514-3p | hsa-miR-1273h-5p | | hsa-miR-6089 | hsa-miR-7162-3p | hsa-miR-4711-3p |
| *circRRP15(4,5)* | up | hsa-miR-30b-3p | hsa-miR-6779-5p | | hsa-miR-1273h-5p | hsa-miR-3689b-3p | hsa-miR-3689c |
| *circZBTB16(2)* | up | hsa-miR-5589-5p | hsa-miR-6764-5p | | hsa-miR-1915-3p | hsa-miR-760 | hsa-miR-3124-3p |
| *ciDGCR8(L12)* | up | hsa-miR-4761-5p | hsa-miR-4731-3p | | hsa-miR-4801 | hsa-miR-4457 | hsa-miR-541-3p |
| *circKIF2A(8,9,10,11)* | up | hsa-miR-659-3p | hsa-miR-202-5p | | hsa-miR-622 | hsa-miR-203b-5p | hsa-miR-6718-5p |
| *circARID4A(6,7,8,9,10,11)* | up | hsa-miR-335-3p | hsa-miR-4778-3p | | hsa-miR-511-5p | hsa-miR-450b-5p | hsa-miR-6809-3p |
| *ciFAM65A(1)* | up | hsa-miR-6862-3p | hsa-miR-6747-3p | | hsa-miR-4729 | hsa-miR-6784-3p | hsa-miR-660-5p |
| *ciMSI2(5)* | up | hsa-miR-7844-5p | hsa-miR-2355-5p | | hsa-miR-3124-3p | hsa-miR-4470 | hsa-miR-888-5p |
| *ciFARP1(2)* | up | hsa-miR-4496 | hsa-miR-6847-5p | | hsa-miR-18a-3p | hsa-miR-3670 | hsa-miR-2117 |
| *circHIBADH(2,3,4)* | up | hsa-miR-181a-2-3p | hsa-miR-4252 | | hsa-miR-192-5p | hsa-miR-215-5p | hsa-miR-548av-3p |
| *circFAM98A(8)* | up | hsa-miR-877-3p | hsa-miR-4701-5p | | hsa-miR-6829-3p | hsa-miR-5193 | hsa-miR-6791-3p |
| *circRDH11(2,3,4,5)* | up | hsa-miR-33a-5p | hsa-miR-20a-3p | | hsa-miR-4686 | hsa-miR-4701-5p | hsa-miR-1265 |
| *circSMARCC1(14,15,16)* | up | hsa-miR-141-5p | hsa-miR-7150 | | hsa-miR-6072 | hsa-miR-4309 | hsa-miR-6773-3p |
| *circZDHHC4(7)* | up | hsa-miR-329-3p | hsa-miR-3941 | | hsa-miR-3974 | hsa-miR-362-3p | hsa-miR-8485 |
| *circGTF2I(7,8,9,10,11)* | up | hsa-miR-12119 | hsa-miR-6884-3p | | hsa-miR-885-5p | hsa-miR-4776-3p | hsa-miR-516a-3p |
| *circZC3HC1(4L,5,6)* | up | hsa-miR-6857-5p | hsa-miR-4691-3p | | hsa-miR-4747-5p | hsa-miR-4533 | hsa-miR-3155a |
| *ciSNORD116-14(1)* | up | hsa-miR-1912-3p | hsa-miR-1295b-5p | | hsa-miR-6888-3p | hsa-miR-3916 | hsa-miR-4685-5p |
| *ciRPL23A(1)* | up | hsa-miR-4746-5p | hsa-miR-874-5p | | hsa-miR-5094 | hsa-miR-34c-3p | hsa-miR-509-3-5p |
| *ciYLPM1(10)* | up | hsa-miR-6507-5p | hsa-miR-548ba | | hsa-miR-548ag | hsa-miR-561-3p | hsa-miR-548ai |
| *ciAGPAT3(1)* | up | hsa-miR-1273h-5p | hsa-miR-1285-3p | | hsa-miR-6884-5p | hsa-miR-5787 | hsa-miR-30b-3p |
| *ciENSG00000297368(2)* | up | hsa-miR-3662 | hsa-miR-3916 | | hsa-miR-3609 | hsa-miR-651-3p | hsa-miR-548n |
| *circDDR1(2,3,4)* | down | hsa-miR-4733-3p | hsa-miR-6085 | | hsa-miR-6766-5p | hsa-miR-7110-5p | hsa-miR-6732-5p |
| *circERGIC3(10,11,12,13)* | down | hsa-miR-2681-3p | hsa-miR-5787 | | hsa-miR-6736-5p | hsa-miR-4270 | hsa-miR-6804-5p |
| *circITFG2(10,11,12S)* | down | hsa-miR-4763-3p | hsa-miR-6756-5p | | hsa-miR-5193 | hsa-miR-6893-5p | hsa-miR-6775-5p |
